# New Perspectives for Fisetin

**DOI:** 10.3389/fchem.2019.00697

**Published:** 2019-10-30

**Authors:** Grzegorz Grynkiewicz, Oleg M. Demchuk

**Affiliations:** Pharmaceutical Research Institute, Warszawa, Poland

**Keywords:** fisetin, flavon-3-ols, synthesis of flavonols, biological activity of flavonols, anti-cancer, anti-aging

## Abstract

Fisetin is a flavonol that shares distinct antioxidant properties with a plethora of other plant polyphenols. Additionally, it exhibits a specific biological activity of considerable interest as regards the protection of functional macromolecules against stress which results in the sustenance of normal cells cytoprotection. Moreover, it shows potential as an anti-inflammatory, chemopreventive, chemotherapeutic and recently also senotherapeutic agent. In view of its prospective applications in healthcare and likely demand for fisetin, methods for its preparation and their suitability for pharmaceutical use are discussed herein.

## Introduction

**Figure 1:**
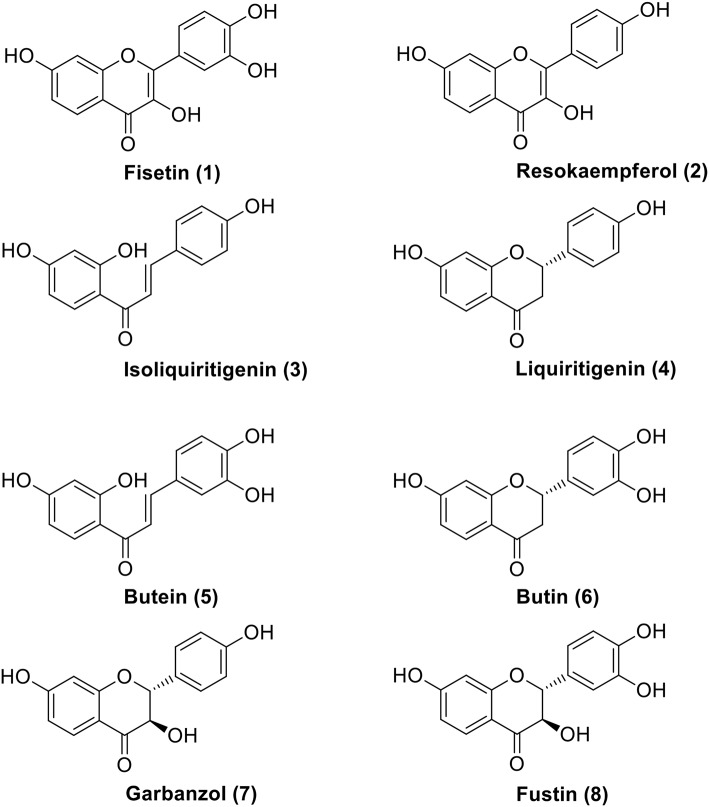
Fisetin (**1**) and biogenetic precursors of the 5-deoxy flavonoid series: chalcones (**3** and **5**), flavanones (**4** and **6**), and dihydroflavonols (**7** and **8**).

The first record of fisetin as an isolate from venetian sumach (*Rhus cotinus* L.) dates back to 1833. A basic chemical characteristics of the compound was provided several decades later by Schmidt (1886), while its structure was elucidated and eventually confirmed by synthesis by S. Kostanecki, who in 1890s started a massive investigation of yellow plant pigments and coined new group names for their sub-categories, presently known as “flavones,” “chromones,” “chalcones,” etc. (Kostanecki et al., 1904). The flavonol fisetin (CAS No. [528-48-3]), conventionally described as: 2-(3,4-dihydroxyphenyl)-3,7-dihydroxy-4H-1-benzopyran-4-one; 3,3′,4′,7-tetrahydroxyflavone; or 5-deoxyquercetin, and represented by the **structural formula 1**, has by now been identified as a secondary metabolite of many plants, occurring in their green parts, fruits, as well as in barks and hardwood (Panche et al., 2016; Hostetler et al., 2017; Verma, 2017; Wang et al., 2018). It was Roux, who in a series of meticulous studies conducted before the advent of modern spectral tools of structural analysis, explained the origin and stereochemistry of oligomeric tannins which contain flavon-3-olic structures closely related to fustin, fisetidinol, fisetin, and similar structures present in various African trees (Roux and Paulus, 1961, 1962; Roux et al., 1961; Drewes and Roux, 1965) (Figure 1). Although condensed tannins used by the leather industry have retained some of their technical significance, today more attention is paid to the presence of fisetin in vegetable constituents of human diet and their role as important epigenetic factors in modulating the state of human health. Fisetin is present in strawberries, apples, persimmons, grapes, onions, kiwi, kale, etc., albeit in low concentration, up to hundreds of micrograms per 1 gram of fresh biomass. The reason for this interest stems from relatively recent observations that compound 1 is not only particularly efficient as an antioxidant agent, but also exhibits remarkable selectivity as regards influencing multiple biological processes considered crucial for biological homeostasis.

These findings naturally raise some questions concerning the general availability of fisetin. Thus far, the natural substance of high chemical purity—high-melting yellow needles, soluble in polar organic solvents and practically insoluble in water—has been available for research purposes as an isolate from plants and as a biochemical reagent which has already become an important molecular probe in human physiology. The question of fisetin's availability naturally arises with the surge in the number of pharmacological studies. Ensuring a uniform quality of the investigated active substance is required when preparing a CTD (Common Technical Document) document necessary before the substance is approved for clinical trials. This question is further discussed in more details.

**Figure 2:**
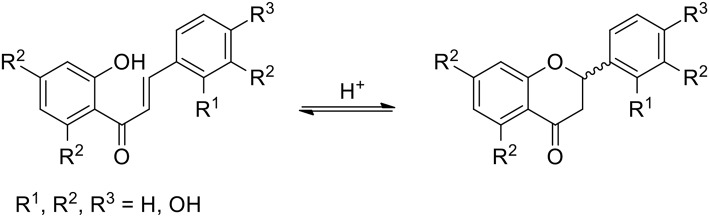
Equilibrium between the chalcone and flavanone counterparts.

Almost all natural phenylpropanoids tend to occur in glycosylated forms, but the glycosides of **1** are seldom mentioned in phytochemical literature, unlike sugar derivatives of its analogs presented in Figure 1. Compounds **2**–**8** are closely related to fisetin: during plant biogenesis chalcones and their isomeric flavanones are subject to two different kinds of hydroxylations (aromatic in the ring B of **4** and alicyclic in the ring C of **6**), both performed by the CYP450 type enzymes. Finally, flavan-ol-3-on-4 (**8**) is oxidized, losing both centers of chirality and affording **1**. The development of a protein fold for the chalcone synthase (CHS, EC 2.3.1.74; and its isomerase CHI, EC 5.5.1.6) constituted a great evolutionary achievement which allowed plants to master a stereoselective phenylpropanoid synthesis and attain many new functions as far as signaling, defense and allelopathy are concerned (Austin and Noel, 2003; Dao et al., 2011; Ngaki et al., 2012; Yin et al., 2018). However, in the abiotic world of chemical synthesis, the position of the isomeric equilibrium between chalcones and their racemic flavanone counterparts can be controlled by a mere change of the pH value (Figure 2) (Pramod et al., 2012; Bhattacharyya and Hatua, 2014; Masesane, 2015). Thus, an interaction of a dietary plant metabolome with human physiology may require special care in interpreting nutritional phenomena, traditionally based on selected marker compounds.

## Chemical Basis for the Selective Biological Activity of Fisetin

**Figure 3:**
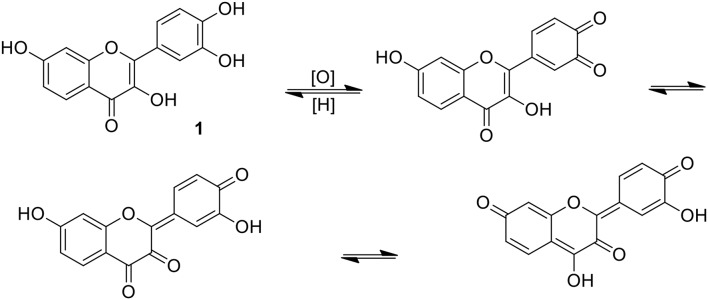
Isomerism of fisetin involving quinone/quinone methide structures; justification of a strongly electrophilic character of the catecholic ring (Awad et al., 2001).

Ample experimental evidences existed to support a simple generalization that practically all plant phenolics exhibit pronounced antioxidant properties (Halliwell, 2006; Galleano et al., 2010; Prior and Wu, 2013). Very complicated chemistry of simple phenolics, comprising the reactivity of free radicals, iono-radicals and organic ionic structures resulting from the proton transfer is in considerable part reflected in their biological activity and pharmacology (Cicerale et al., 2008; Pereira et al., 2009; Baruah, 2011; Adeboye et al., 2014). Polyphenolic structures extended by the inclusion of a catechol ring are particularly susceptible to specific aromatic electron delocalization which may involve, as a result of contact with the hydrogen acceptors, quinone, and vicinal diketone structures, as exemplified for 1 in Figure 3 (Awad et al., 2001). Apparently, such intermediates are less prone to flavonoid oligomerization, but can be active as acceptors of a variety of cellular nucleophiles.

## Cellular Senescence and Fisetin

Nearly six decades ago the phenomenon of a finite proliferation capacity of human fibroblasts was discovered (Hayflick, 1965, 1974) initiating a period of extensive studies on cell growth arrest mechanisms, particularly in connection to the causes of the aging process. According to the recent findings, cellular senescence which is essentially permanent, appears to play distinct roles both: in normal physiology and various pathologies. Senescent cell phenotypes, which normally secrete inflammatory proteins (SASP) and aim at apoptosis, can undergo certain modes of pharmacologically induced intervention leading to the cell fate reversal (Kuilman et al., 2010, p. 92). Essentially, senescence and cancerogenesis (oncogenesis) direct cell fate into opposite directions, which is of crucial importance when it comes to understanding the mechanisms of chemotherapy during which tumor regression can result from the induced senescence response (Campisi, 2013; van Deursen, 2014; Mendelsohn et al., 2015). Despite the fact that the senescent cells can also undergo cancer promotion and progression, influence of pharmacological agents on both reverse processes will remain an important field of research for a long time. At present both: the idea of senescence eliciting stimuli under a variety of stressful conditions and the ability to counteract and/or reverse the senescence-associated secretory phenotype are strongly interconnected. This is based on the theories of aging which point to the detrimental effects of reactive oxygen species (ROS), either of mitochondrial origin or generated by an environmental impact (Gil del Valle, 2011, p. 102; Liochev, 2013). While the notion of natural products, particularly those ingested with diet, as protectors against ROS, is already well-established on the cellular level, it seems too general to explain in detail particular selective activities of a myriad of plant secondary metabolites for whom claims of beneficiary medicinal effects have already been formulated.

Apart from the antibiotical activity (Manjolin et al., 2013; Borsari et al., 2016), fisetin shares a distinct antioxidating activity with many other polyphenolic compounds, which was confirmed by various *in vitro* as well as *in vivo* models (Khan et al., 2013; Lall et al., 2016; Jiang et al., 2018; Kashyap et al., 2018). Additionally, antioxidant effects of **1** and in particular the induction of the glutathione synthesis are considered important as far as neuroprotection is concerned.

Also, much attention has been paid to the anticancer activity of **1**. *In vitro* studies were performed which offer a panoramic view of the target organ selectivities, as well as an overview of the macromolecular targets. The latter include: AMP-activated protein kinase (AMPK); cyclooygensae (COX); epidermal growth factor receptor (EGFR); extracellular signal-regulated kinase (ERKI1/2); matri metalloproteinase (MMP); nuclear factor-kappa B (NF-κB); prostate-specific antigen (PSA) transcription factor T-cell factor (TCF); TNF-related apoptosis-inducing ligand (TRAIL); Wnt inhibitory factor (WIF-1); X-linked inhibitor of apoptosis (XIAP), among others (Lall et al., 2016; Hostetler et al., 2017; Kashyap et al., 2018; Wang et al., 2018).

The anticancer activity of fisetin can be enhanced by some auxiliary substances. For example, fisetin significantly impairs carcinoma cell growth in the presence of ascorbic acid, which results in a 61% inhibition of cell growth, in 72 h; the treatment with ascorbic acid alone had no effect on cellular proliferation (Kandaswami et al., 1993). It was also shown that flavonols of the fisetin type extracted from Allium Vegetables, may play a role of such an auxiliary in combination with well-defined anticancer drugs and enhance the antiproliferative activity of cis-diamminedichloroplatinum(II), nitrogen mustard, and busulphan in human tumor cell culture systems. The analysis of the chemical composition of the flavonol extracts from different kinds of Allium Vegetables and their effects on the neoplastic transformation of NIH/3T3 cells has already been presented (Leighton et al., 1992).

Other activities along this line include: enhancement of the long-term memory, antidepressant effects, inhibition of ischemic reperfusion injury and amelioration of behavioral deficits following a stroke (Khan et al., 2013; Maher, 2015; Currais et al., 2018; Kashyap et al., 2018).

Perhaps the most promising of the documented fisetin biological activities resides in the anticipated possibility of targeting fundamental aging mechanisms. Although the senescent cells resist apoptosis through upregulation of the senescent-cell anti-apoptotic pathways (SCAP), it has been demonstrated that some combination of pharmacological agents (called senolitics or senotherapeutics; e.g., Dasatinib with Quercetin) can overcome this resistance. A follow-up screening of the flavonoids revealed that 1 was even more effective than quercetin and could accomplish the task of reducing senescence markers as a single agent (Yousefzadeh et al., 2018). Model experiments that started with S. cerevisiae and proceeded through D. melanogaster all the way to vertebrate animals clearly demonstrate that fisetin is able to extend the lifespan of investigated organisms of both sexes (Wood et al., 2004; Si et al., 2011; Wagner et al., 2015). As a result of these findings J. L. Kirkland's team at the Mayo Clinic has recently designed and begun a clinical trial aimed at the “Alleviation by Fisetin of Frailty, Inflammation, and Related Measures in Older Adults” (AFFIRM-LITE) with fisetin administered orally in doses up to 20 mg per kilogram of patient body weight^1^. In view of poor solubility (10.45 μg/mL), relatively low oral bioavailability (44%) and rapid metabolism, such a development warrants interest in the prospective fisetin sources for suitable pharmaceutical formulations.

Recent *in vitro* studies have given a mechanistic insight into how fisetin inhibits the target of the rapamycin pathway in various cell models and therefore influences cellular pathways that are known to affect aging (Syed et al., 2013; Pallauf et al., 2016).

It has also been found that fisetin in combination with other epigenetically active molecules which are able to cross the blood-aqueous and blood-retina barriers exhibit synergistic beneficial effects. This applies for a low dose red wine polyphenols, as well as for vitamin D3 and some other compounds of small molecular weight, synergistically improving visual acuity in patients with advanced atrophic age-related muscular degeneration, including the older ones with advanced stages of the disease for whom very few options remained (Ivanova et al., 2017).

Taking into account moderate international market availability of natural fisetin on the one hand and its high biological activity on the other hand, food supplementation of that compound is still rare. On the market there are several dietary supplements containing fisetin which according to the producers have “apparent brain-health benefits.” They are advertised as seno-therapeutic (Yousefzadeh et al., 2018), anticarcinogenic, dietary antioxidants for Health Promotion (Khan et al., 2013), as neurotrophic, anti-inflammatory agents that “may help fine-tune your mind,” as well as “help promote cognition and overall brain health,” or “help patients with Alzheimer's and Parkinson's disease.” At the same time the producers shake off all responsibility for the product by adding a disclosure to the effect: “These statements have not been evaluated by the Food and Drug Administration. This product is not intended to diagnose, treat, cure or prevent any disease.” Since the majorities of studies on biological activity of fisetin are mainly academic, clinical trials evaluating its activity are still rare. Recent clinical trials^2^ have provided a detailed evaluation of fisetin's anti-oxidative, anti-apoptotic, hyperglycemia alleviating, kidney function enhancing effects. Studies on altering biologic markers of inflammation, insulin resistance, and bone resorption and frailty in older postmenopausal women (AFFIRM) have also been performed.

Recently, in connection with the pharmacokinetic study of 1 performed on Sprague-Dawley rats the presence of 3′-O-methylated metabolite (geraldol) was recorded along with sulfates and glucuronides. Subsequently, a suggestion was advanced that this transformation is advantageous, since it renders 1 more stable, as evidenced by higher AUC concentrations and better distribution to distal organs, including the brain, when compared to other metabolites (Mehta et al., 2018).

In addition to age and oncology related diseases it was also indicated that iron complexes with fisetin derivatives have a biological effect similar to that of desferrioxamine available on the market in oral applications for the treatment of β-thalassemia (Yildiz et al., 2010).

## Quest for the Fisetin API

According to current estimates, strawberries, with 160 μg/g, are the richest source of fisetin, which makes the prospect of its isolation in the technical manufacturing process poor, despite the fact that the fruits belong to industrial scale agricultural crops. In order to recover **1** from a fruit a very selective solid phase extraction process using appropriate synthetic resins would be required, producing significant amounts of processing water waste. As we have learned in the past decades from Active Pharmaceutical Ingredient (API) manufacturing, their processes evolve continuously. Thus, medicinal products of natural origin often undergo a semi-synthetic or synthetic process, before they are defined as a biotechnological product. Such transitions stem from current general indications which strongly advocate green chemistry and environmental protection in technical process design, while complete development of biotechnological process usually require long period of time (Fox et al., 2007; Sheldon, 2008, p. 193; Patel, 2018; Sun et al., 2018). Some efforts toward designing a biosynthetic pathway to fisetin from L-tyrosine present in Escherichia coli and Saccharomyces cerrevisiae have already been reported (Jendresen et al., 2015; Stahlhut et al., 2015; Jones et al., 2016; Pandey et al., 2016; Rodriguez et al., 2017). Nevertheless, in the case of a molecule so small and simple as 1, the synthetic stage of API manufacturing is imminent, which calls for a critical evaluation of the already described syntheses, especially in view of the current requirements for pharmaceutical GMP and quality assurance.

**Figure 4:**
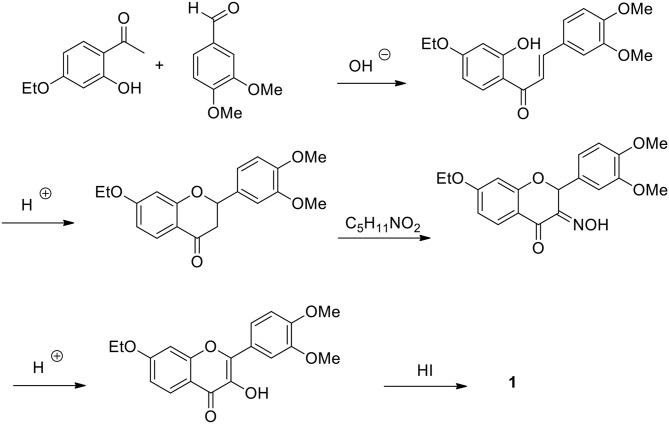
Kostanecki's synthesis of fisetin.

The first synthesis of **1**, completed in 1904 (Kostanecki et al., 1904), involved the preparation of partially protected chalcone which could be cyclized to flavanone under acidic conditions. The next step in the advancement of phenylpropanoid intermediate oxidation was achieved by amyl nitrate which served as an oxidation agent. Stepwise oxime hydrolysis and alkylated phenol groups deprotection by HI afforded fisetin identical with the authentic sample isolated from the plant source (Figure 4). This method has several recent modifications mostly devoted to the oxidation and demethylation steps (Hasan et al., 2010; Borsari et al., 2016).

**Figure 5:**
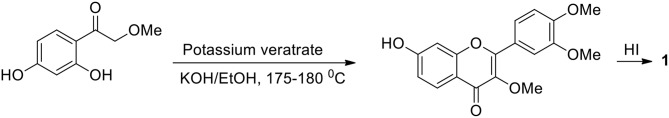
Robinson's synthesis of fisetin.

The next attempt at preparing **1** was made by Robinson in 1926 (Allan and Robinson, 1926). The treatment of ω-methoxyresacetophenone with veratric anhydride in the presence of potassium veratrate in ethanol in a sealed glass tube at 180°C afforded required chromen-4-one which was converted to **1** by hydrogen iodide (Figure 5).

**Figure 6:**
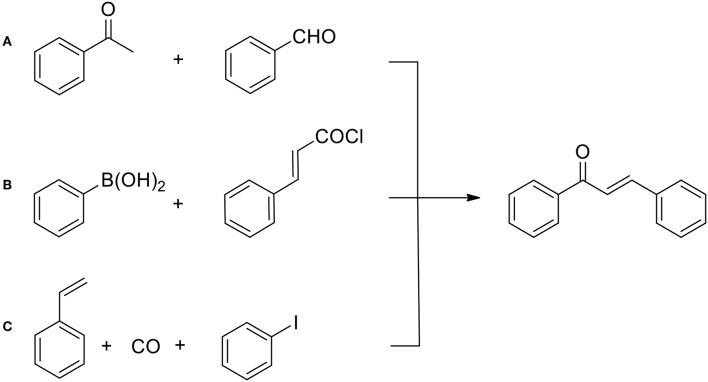
General methods for the chalcone preparation: **(A)** Claisen-Schmidt condensation (base catalyzed); **(B)** Suzuki cross-coupling (Pd catalyzed); **(C)** carbonylative Heck olefin arylation (catalyzed by Pd complexes).

Recently, more friendly methods have been developed for flavonoids in general and flavonols in particular. It should be pointed out that currently, as illustrated on Figure 6, there exists a wide selection of synthetic methods used to prepare chalcones which remain principal intermediates for cyclization to chromanones (Zhuang et al., 2017). In particular, with the aid of modern transition metal catalysts, the formation of carbon—carbon bonds between two aromatic synthons can take place in a variety of ways, as discovered by Heck, Suzuki and Negishi (Johansson-Seechurn et al., 2012).

**Figure 7:**
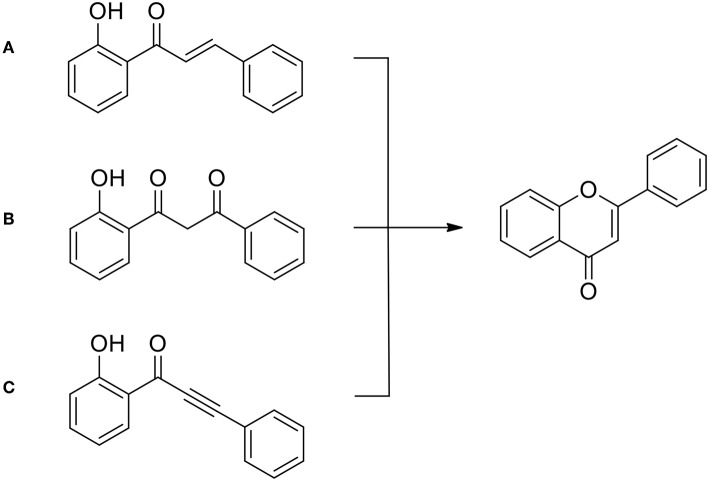
General approaches to flavone syntheses: **(A)** from chalcones (following Kostanecki's synthesis); **(B)** from 1,3-diaryl propandiones (following Kostanecki—Robinson—Venkataraman ideas); **(C)** from phenylalkenyl ketones, according to Lee (2017).

Chalcones hydroxylated in the ortho- position to the ketone group are of special interest here, because they can easily undergo cyclization leading to the flavone precursors and flavones (Figure 7), much more seldom to aurones (not shown) (Krohn et al., 2009; Megens and Roelfes, 2012; Nising and Bräse, 2012; Zhang et al., 2013; Masesane, 2015).

**Figure 8:**
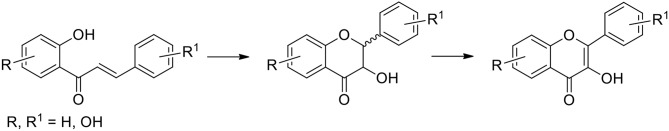
General scheme for the AFO synthesis of flavonols upon chalcone epoxidation with H_2_O_2_.

Considering facile availability of chalcones (easily transformable to flavones, for example by iodine promoted cyclization conducted in DMSO), their epoxidation followed by an intramolecular oxirane ring opening could be considered as the method of choice for flavonol preparation. Indeed, such a pathway was developed into a practical synthetic method by successive efforts of Irish and Japanese researchers and their followers. Currently known as the Algar-Flynn-Oyamada reaction (AFO), it uses the basic solution of hydrogen peroxide as a crucial reagent (Oyamada, 1935; Gunduz et al., 2012; Bhattacharyya and Hatua, 2014; Shen et al., 2017). Its general scheme, indicating typical substitution patterns, is presented below (Figure 8). This reaction offers a possibility of the aurone product formation by the α-oxirane ring opening, with only moderate yields of flavonols usually reported. It should be mentioned that flavones which are more readily available than flavonols by a variety of preparative procedures can be easily halogenated in position 3 using reagents that generate positively charged halogen atoms, such as NCS (*N*-chlorosuccinimid), NBS (*N*-bromosuccinimid), or iodine in the presence of CAN (cerium-ammonium nitrite). Apparently, this seemingly obvious avenue has not been exploited as a practical method for preparing flavonols.

**Figure 9:**
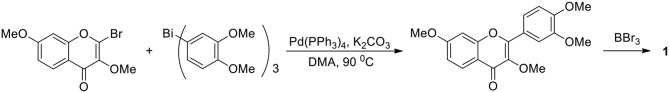
Synthesis of fisetin by arylation of the 2-bromochromone derivative.

In a more recent attempt at the preparation of flavonols organometallic chemistry was applied to the 2-bromochromanone Pd catalyzed arylation step, as illustrated below (Figure 9). In the case of fisetin two crucial steps of synthesis were completed in 75% of the overall yield (Rao and Kumar, 2014). In principle, three equivalents of the bromochromone substrate could be arylated by one equivalent of an appropriate phenylbismuth reagent in such a reaction.

It seems that the initial idea of Kostanecki, where flavanones were chosen as the principal substrates for the transformation, has not been fully exploited yet, although it has already been demonstrated that precursors such as flavones can be directly oxidized to flavanols, for example by 3,3-dimethyldioxirane (Maloney and Hecht, 2005). In this connection, a semi-synthesis should be mentioned as more than a theoretical possibility. The example of hesperidin's (abundant citrus flavanone easily recoverable from orange peels) transformation into methoxylated 3-flavonol in the 5 synthetic steps clearly indicates that some natural products can be treated as suitable substrates toward the required flavonoid material (Garg et al., 2001; Lewin et al., 2010).

While the above list of reactions seems to exhaust the chemical synthetic means for prospective fisetin API availability (Molga et al., 2019), current industrial trends indicate that biotransformations are to be considered an ultimate resource of chemical entities for human use in food and medicine supplements. To this end, substantial knowledge concerning fisetin biosynthesis exists: chalcone isoliquiritigenin is cyclized to flavanone liquiritigenin, hydroxylased to catechin garbanzol, flavone resokaempferol, and oxidized to **1**. All the biocatalysts for this chain of transformations are known, moreover, they have been successfully expressed in microorganisms for the preparation of both quercetin and fisetin (Jendresen et al., 2015; Stahlhut et al., 2015; Jones et al., 2016; Pandey et al., 2016; Rodriguez et al., 2017).

## Conclusions and Outlook

The average daily intake of fisetin from various vegetable sources is estimated to be at the level of 0.4 mg (Kashyap et al., 2018). In view of recent findings concerning its beneficial antioxidant, anti-inflammatory, antitumor, neuroprotective, and anti-aging biological activities, a growing need for a high purity substance fit for pharmaceutical development can be forecasted. The quest for the medicinal status of 1 may be slow and difficult, as the history of flavonoids' retraction from the vitamin status shows. Nevertheless, current demand for natural products such as fisetin may come from the less regulated markets, as in the case of functional food or dietary supplements. There is no uniform legal concept for functional food and its current definition: “natural or processed foods that contain biologically-active compounds; which, in defined, effective, non-toxic amounts, provide a clinically proven and documented health benefit utilizing specific biomarkers, for the prevention, management, or treatment of a chronic disease or its symptoms” (Danik and Jaishree, 2015; Martirosyan, 2015) may not sound ideal. Nevertheless, it serves the purpose in terms of health claim use, and it can certainly promote new market entries, provided good science is used to support the presence of new constituents in the food products. Chemical synthesis seems to be an obvious first aid solution, with the process design based on chalcone intermediates, along the AFO route. However, this simple chemistry requires considerable optimization efforts aimed at minimalization or even elimination of the protective group chemistry input. Alternatively, availability of suitable (i.e., 5-deoxy) intermediate raw materials should be carefully examined, since flavon-3-ols can be obtained by chemical transformation from their structural relatives such as flavan-4-ones, flavones, catechins and chalcones. In any case, care should be taken to enhance poor solubility and bioavalability of 1. Some technical solutions have already been proposed (DeCorte, 2016; Chadha et al., 2019). The issue of fisetin's low solubility could be overridden by way of its complexation with cyclosophoroase dimer and cyclodextrins which also significantly improves the cytotoxicity of fisetin against HeLa cells (Jeong et al., 2013; Zhang et al., 2015). Such studies may well serve to extend the medicinal chemistry capacity of 1 as well as its analogs and derivatives, following numerous examples of secondary metabolites exploited as drug leads. Finally, it is likely that the future of fisetin manufacturing as an API (or its precursor) might lie in the realm of biotechnology (Wu et al., 2018; Huccetogullari et al., 2019; Mark et al., 2019). In any case, it should be pointed out that a single agent (such as **1**) supplementation may bring about different overall pharmacological effects than a vegetable diet rich in the same substance, since in the latter case a whole 5-deoxy flavonoid segment of a plant metabolome (which comprises many related individual chemicals) collides with human system biology, leading to a considerably more complex network of mutual interactions.

## Author Contributions

The authors have an equal contribution in the conceptualisation, data collection, and manuscript preparation.

### Conflict of Interest

The authors declare that the research was conducted in the absence of any commercial or financial relationships that could be construed as a potential conflict of interest.

## Footnotes

^1^ClinicalTrials.gov Identifier: NCT03675724.

^2^ClinicalTrials.gov Identifier: NCT03430037.
